# Single‐Molecule Conductance Studies of Organometallic Complexes Bearing 3‐Thienyl Contacting Groups

**DOI:** 10.1002/chem.201604565

**Published:** 2017-01-16

**Authors:** Sören Bock, Oday A. Al‐Owaedi, Samantha G. Eaves, David C. Milan, Mario Lemmer, Brian W. Skelton, Henrry M. Osorio, Richard J. Nichols, Simon J. Higgins, Pilar Cea, Nicholas J. Long, Tim Albrecht, Santiago Martín, Colin J. Lambert, Paul J. Low

**Affiliations:** ^1^ School of Chemistry and BiochemistryUniversity of Western Australia35 Stirling HighwayCrawley6009WAAustralia; ^2^ Department of PhysicsLancaster UniversityLancasterLA1 4YBUK; ^3^ Department of Laser Physics, Women Faculty of ScienceBabylon UniversityIraq; ^4^ Department of ChemistryDurham UniversitySouth Rd.DurhamDH1 3LEUK; ^5^ Department of ChemistryUniversity of LiverpoolCrown St.LiverpoolL69 7ZDUK; ^6^ Department of ChemistryImperial College LondonLondonSW7 2AZUK; ^7^ Centre for Microscopy, Characterisation and AnalysisUniversity of Western AustraliaCrawleyWestern Australia6009Australia; ^8^ Departamento de Química Física, Facultad de CienciasUniversidad de Zaragoza50009ZaragozaSpain; ^9^ Instituto de Nanociencia de Aragón (INA) y Laboratorio de Microscopias, Avanzadas (LMA), Edificio I+D Campus Rio EbroUniversidad de ZaragozaC/Mariano Esquillor, s/n50018ZaragozaSpain; ^10^ Departamento de FísicaEscuela Politécnica NacionalAv. Ladrón de Guevara, E11-253170525QuitoEcuador; ^11^ Instituto de Ciencias de Materiales de Aragón (ICMA)Universidad de Zaragoza-CSIC50009ZaragozaSpain

**Keywords:** contacting group, density functional calculation, organometallic chemistry, scanning tunneling microscopy, single-molecule conductors

## Abstract

The compounds and complexes 1,4‐C_6_H_4_(C≡C‐*cyclo*‐3‐C_4_H_3_S)_2_ (**2**), *trans*‐[Pt(C≡C‐*cyclo*‐3‐C_4_H_3_S)_2_(PEt_3_)_2_] (**3**), *trans*‐[Ru(C≡C‐*cyclo*‐3‐C_4_H_3_S)_2_(dppe)_2_] (**4**; dppe=1,2‐bis(diphenylphosphino)ethane) and *trans*‐[Ru(C≡C‐*cyclo*‐3‐C_4_H_3_S)_2_{P(OEt)_3_}_4_] (**5**) featuring the 3‐thienyl moiety as a surface contacting group for gold electrodes have been prepared, crystallographically characterised in the case of **3**–**5** and studied in metal|molecule|metal junctions by using both scanning tunnelling microscope break‐junction (STM‐BJ) and STM‐*I*(*s*) methods (measuring the tunnelling current (*I*) as a function of distance (*s*)). The compounds exhibit similar conductance profiles, with a low conductance feature being more readily identified by STM‐*I*(*s*) methods, and a higher feature by the STM‐BJ method. The lower conductance feature was further characterised by analysis using an unsupervised, automated multi‐parameter vector classification (MPVC) of the conductance traces. The combination of similarly structured HOMOs and non‐resonant tunnelling mechanism accounts for the remarkably similar conductance values across the chemically distinct members of the family **2**–**5**.

## Introduction

The development of a range of complementary and relatively facile methods for the measurement of the electrical properties of single molecules has seen a renaissance in the field of molecular electronics.^1^, ^2^, ^3^, ^4^ The continued progress of the area from fundamental science towards technology now rests on a number of key issues,^5^ among which are the reliable contacting of molecules within a junction,^6^, ^7^ the reduction in electronic variation between individual junctions^1^, ^8^, ^9^, ^10^, ^11^, ^12^ and the optimisation of the transport properties of these junctions.^13^, ^14^, ^15^, ^16^ To these ends, considerable effort is being made to explore the effects of the electrode–molecule contact groups and structure of the contact,^17^, ^18^, ^19^, ^20^ the potential applications of non‐metallic electrodes to create an “all‐carbon” molecular electronic device platform,^21^ as well as the backbone structure of the molecular component on the electrical properties of the junction.^22^, ^23^, ^24^

Although the majority of single‐molecule and thin‐film junctions studied to date have been based on organic molecules, such as alkanes,^25^, ^26^ oligo(arylene)ethynylenes^27^, ^28^, ^29^ and polyynes,^30^, ^31^, ^32^, ^33^ metal complexes have also been recognised as potential components in a future molecular electronics technology.^34^, ^35^, ^36^, ^37^, ^38^ Metal complexes offer a range of potential advantages over structurally and electronically simpler organic molecules, including redox activity and a wider range of readily accessible and systematically variable spin‐states and magnetic properties,^39^, ^40^, ^41^, ^42^ diversity of molecular structure and potential for modular construction through in situ or “on surface” coordination chemistry,^43^, ^44^, ^45^, ^46^ better alignment of the frontier molecular orbitals with the Fermi level of the (usually metallic) junction electrodes,^8^, ^47^, ^48^, ^49^, ^50^ as well as high thermoelectric efficiency.^51^

In some earlier studies of organometallic complexes in molecular electronics, the complex *trans*‐[Pt(C≡CC_6_H_4_SAc)_2_(PPh_3_)_2_] was assembled within a mechanically controlled break‐junction (MCBJ) based molecular junction. From the resulting *I*/*V* curves, collected over a bias range of ±5 V, a resistance of 5–50 GΩ (i.e., *G*=0.2–0.02 nS; 20–2×10^−6^ G_0_) was estimated at the extremes of the bias range, some three orders of magnitude less conductive than similarly contacted organic oligoarylene systems.^52^ This “insulating” behaviour, even under such a high applied bias, was ascribed to the largely σ‐type Pt−C(sp) bonds in the C≡C−Pt−C≡C backbone, although it is clear that at this bias voltage, the conductance mechanism is likely to be field emission rather than tunnelling.^53^ In contrast, a later study with a family of complexes of type *trans*‐[Pt(C≡CC_6_H_4_SAc)_2_(L)_2_] (L=PCy_3_, PPh_3_, P(OEt)_3_) in crossed‐wire junctions at more modest bias (up to 1 V) revealed a two‐ to threefold higher conductance than 1,4‐(4‐AcSC_6_H_4_C≡C)_2_C_6_H_4_, which was ascribed to the shorter sulfur–sulfur distance in the metal complexes.^54^

In seeking to enhance the wire‐like response, significant attention was turned to ruthenium bis(alkynyl) complexes, which are generally thought to offer more significant d–π orbital mixing in the occupied frontier molecular orbitals.^9^, ^34^, ^50^, ^55^, ^56^, ^57^ The thioacetate complex *trans*‐[Ru(C≡CC_6_H_4_SAc‐4)_2_(dppm)_2_] (dppm=1,1‐bis(diphenylphosphino)methane) has been assembled into monolayers and studied within a scanning tunnelling microscope break junction (STM‐BJ), with a comparison made to the oligo(phenyleneethynylene) (OPE) compound 1,4‐(4‐AcSC_6_H_4_C≡C)_2_C_6_H_4_ as a benchmark. Extrapolation to single‐molecule conductances gave values of 19±7 nS (ca. 2.5×10^−4^ G_0_) for the ruthenium complex and 3.6±2.0 nS (ca. 4.6×10^−5^ G_0_) for the OPE. The higher conductance has been attributed to both the shorter molecular length and the extensive Ru(d)−C≡C(π) mixing in the metal complex.^56^ These concepts have been extended to other examples of organometallic wires based on group 8 metal centres and the *trans*‐bis(alkynyl) motif, with topics of interest including the exploration of surface contacting groups,^9^, ^50^ the inclusion of multiple metal centres along the molecular back‐bone^35^, ^47^, ^57^ and electronic function beyond that of a simple wire, such as charge storage and gated transistor‐like response.^41^, ^58^

One particular advantage of organometallic complexes within the field of molecular electronics lies in the ability to systematically alter the molecular structures of these systems with a fair degree of synthetic ease, which permits a modular approach to molecular designs and a systematic search for structure–property relationships. In seeking to further explore the electrical properties of oligophenyleneethynylene (OPE), and *trans*‐bis(alkynyl) complexes of platinum and ruthenium, we have turned to such a systematic study here. Here, the 3‐thienyl moiety,^59^, ^60^, ^61^ which is readily introduced into both organic and organometallic structures, is used as a contacting group for the ready attachment of organic, ruthenium and platinum‐based organometallic complexes within Au|molecule|Au junctions and electrical characterisation by using both the *I*(*s*)^62^ (measuring the tunnelling current (*I*) as a function of distance (*s*)) and STM‐BJ^63^ methods. The conductance results are interpreted with the aid of DFT level calculations and junction simulations.

## Results and Discussion

### Synthesis and characterisation

**Scheme 1:**
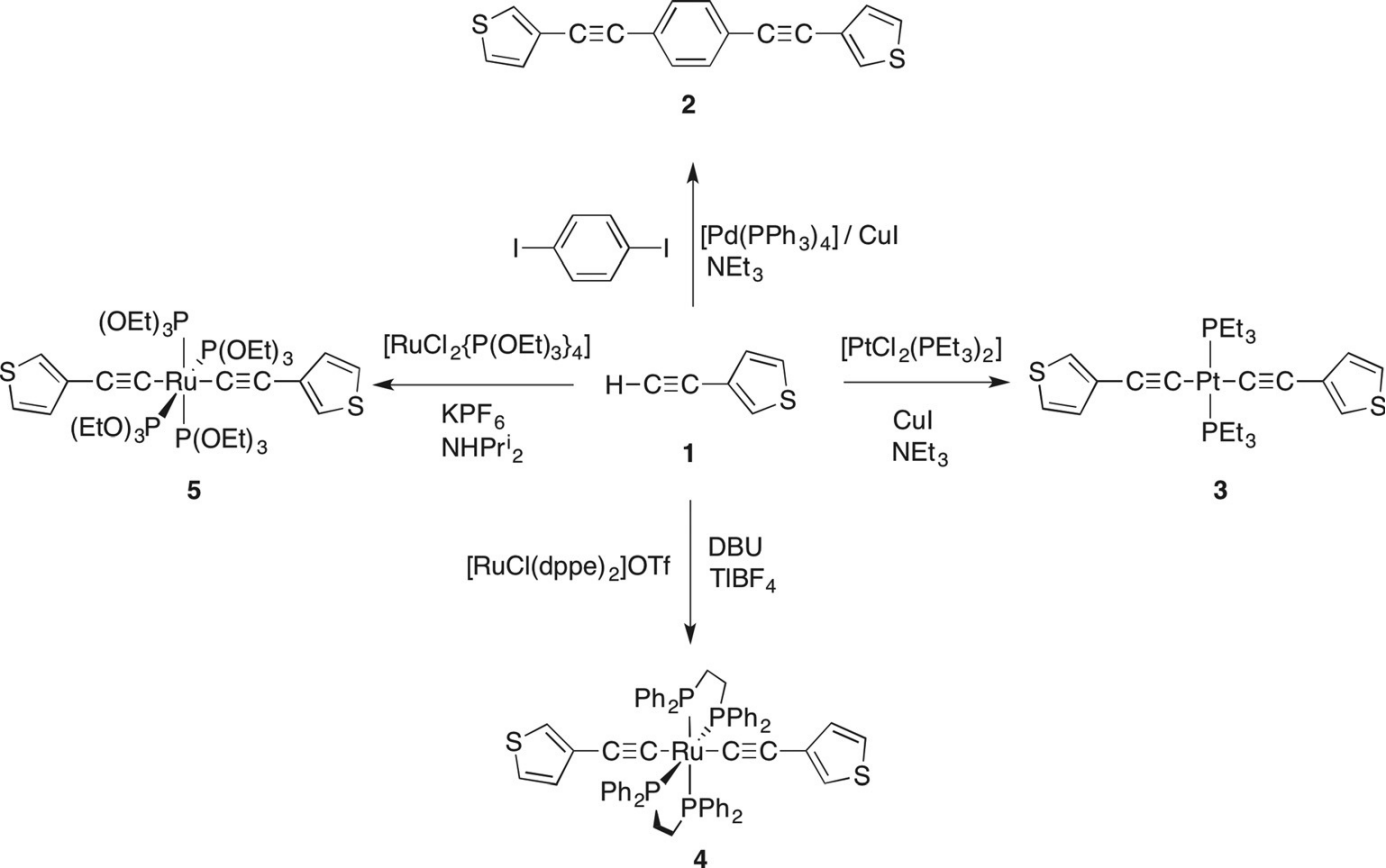
The preparation of compounds **2**–**5**.

The parent alkyne 3‐ethynyl thiophene (HC≡C‐*cyclo*‐3‐C_4_H_3_S, **1**) was obtained from the Sonogashira cross‐coupling of 3‐bromothiophene with trimethylsilylacetylene and subsequent deprotection.^59^ Further cross‐coupling of **1** with 1,4‐diiodobenzene gave 1,4‐C_6_H_4_(C≡C‐*cyclo*‐3‐C_4_H_3_S)_2_ (**2**; Scheme 1).^59^ The metal complex *trans*‐[Pt(C≡C‐*cyclo*‐3‐C_4_H_3_S)_2_(PEt_3_)_2_] (**3**) was prepared from the Cu^I^‐catalysed reaction of **1** and [PtCl_2_(PEt_3_)_2_] in NEt_3_,^64^ whilst *trans*‐[Ru(C≡C‐*cyclo*‐3‐C_4_H_3_S)_2_(dppe)_2_] (**4**; dppe=1,2‐bis(diphenylphosphino)ethane) was obtained from the one‐pot reaction of [RuCl(dppe)_2_]OTf with **1**, in the presence of either KO*t*Bu or 1,8‐diazabicyclo[5.4.0]undec‐7‐ene (DBU) and TlBF_4_.^65^ Complexes of the general form *trans*‐[Ru(C≡CR)_2_{P(OEt_3_)_3_}_4_] have been prepared in moderate yield from reaction of *trans*‐[RuCl_2_{P(OEt)_3_}_4_] with an excess of LiC≡CR.^66^ The complex *trans*‐[Ru(C≡C‐*cyclo*‐3‐C_4_H_3_S)_2_{P(OEt)_3_}_4_] (**5**) was obtained here simply by allowing the reaction of *trans*‐[RuCl_2_{P(OEt)_3_}_4_]^67^ with **1**, KPF_6_ and NH*i*Pr_2_ in ethanol to proceed for 12 days at room temperature, with isolation of the desired compound being achieved by precipitation from methanol. The long reaction time was compensated by the simple reaction conditions and work‐up, compatibility with alkynes substituted with sensitive functional groups and improved yields. The compounds were each characterised by the usual array of ^1^H, ^13^C{^1^H} and, in the case of **2**–**5**, ^31^P{^1^H} NMR spectroscopies, mass spectrometry and elemental analysis.

### Molecular structures

**Figure 1:**
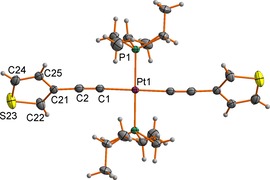
A plot of a molecule of *trans*‐[Pt(C≡C‐*cyclo*‐3‐C_4_H_3_S)_2_(PEt_3_)_2_] (**3**) (ellipsoids drawn at the 50 % probability level) showing the atom labelling scheme. Bond lengths [Å]: Pt(1)−P(1) 2.3022(5); Pt(1)−C(1) 2.007(2); C(1)−C(2) 1.185(3); C(2)−C(21) 1.452(3); C(21)−C(22) 1.373(3); C(22)−S(23) 1.705(3); S(23)−C(24) 1.682(3); C(24)−C(25) 1.393(3); C(21)−C(25) 1.428(3). Bond angles [°]: P(1)‐Pt(1)‐C(1) 88.32(6), 91.68(6); Pt(1)‐C(1)‐C(2) 177.83(19); C(1)‐C(2)‐C(21) 177.3(2).

**Figure 2:**
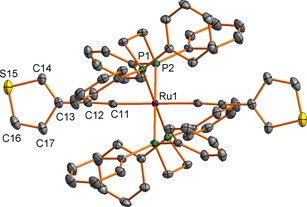
A plot of one molecule of *trans*‐[Ru(C≡C‐*cyclo*‐3‐C_4_H_3_S)_2_(dppe)_2_] (**4**) (ellipsoids drawn at the 50 % probability level) showing the atom labelling scheme. Bond lengths (molecule 1, Å): Ru(1)−P(1) 2.3539(5); Ru(1)−P(2) 2.3602(5); Ru(1)−C(11) 2.0611(19); C(11)−C(12) 1.218(3); C(12)−C(13) 1.433(3); C(13)−C(14) 1.373(3); C(14)−S(15) 1.709(3); S(15)−C(16) 1.709(3); C(16)−C(17) 1.364(3); C(13)−C(17) 1.448(3). Bond angles (molecule 1, °): P(1)‐Ru(1)‐P(2) 82.90(2), 97.10(2); P(1)‐Ru(1)‐C(11) 94.38(5), 85.62; P(2)‐Ru(1)‐C(11) 97.28(5), 82.72(5); Ru(1)‐C(11)‐C(12) 176.2(2); C(11)‐C(12)‐C(13) 176.1(2). Bond lengths (molecule 2, Å): Ru(2)−P(3) 2.3464(4); Ru(2)−P(4) 2.3586(4); Ru(2)−C(21) 2.0626(18); C(21)−C(22) 1.217(3); C(22)−C(23) 1.431(3); C(23)−C(24) 1.389(10); C(24)−S(25) 1.715(10); S(25)−C(26) 1.673(8); C(26)−C(27) 1.352(12); C(23)−C(27) 1.411(10). Bond angles (molecule 2, °): P(3)‐Ru(2)‐P(4) 97.86(2), 82.14(2); P(3)‐Ru(2)‐C(21) 96.61(5), 83.39(5); P(4)‐Ru(1)‐C(21) 84.93(5), 95.07(5); Ru(2)‐C(21)‐C(22) 177.3(2); C(21)‐C(22)‐C(23) 176.0(2).

**Figure 3:**
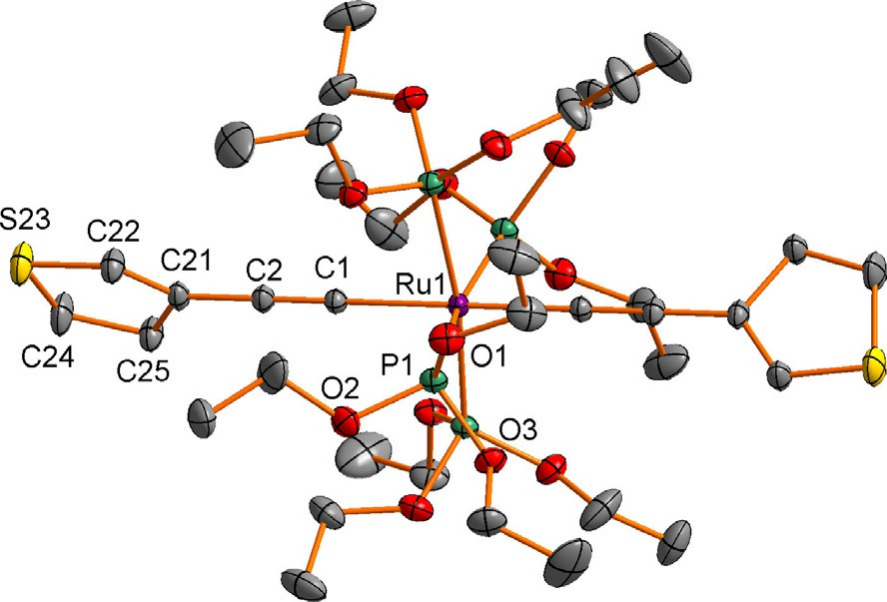
A plot of a molecule (ellipsoids drawn at the 30 % probability level) of *trans*‐[Ru(C≡C‐*cyclo*‐3‐C_4_H_3_S)_2_{P(OEt)_3_}_4_] (**5**) showing the atom labelling scheme. Bond lengths [Å]: P(1)−Ru(1) 2.3149(3); Ru(1)−C(1) 2.0592(15); C(1)−C(2) 1.221(2); C(2)−C(21) 1.421(2); C(21)−C(22) 1.36(2); C(22)−S(23) 1.599(12); S(23)−C(24) 1.741(7); C(24)−C(25) 1.480(11); C(25)−C(21) 1.45(2). Bond angles [°]: P(1)‐Ru(1)‐P(1) 90.338(2), 171.19(2); P(1)‐Ru(1)‐C(1) 94.40(1), 85.60(1); Ru(1)‐C(1)‐C(2) 180; C(1)‐C(2)‐C(21) 180.

Single crystals of **3**, **4** and **5** suitable for X‐ray diffraction were obtained by recrystallisation by slow diffusion of hexanes (**3**, **4**) or EtOH (**5**) into CH_2_Cl_2_ solutions of the complexes. Plots of the molecules showing the atom labelling schemes are given in Figures 1, Figures 2 and Figures 3 and the important bond lengths and angles are summarised in the relevant figure captions.

In the crystal, **3** is situated on a crystallographic inversion centre. The platinum atom shows the expected square‐planar geometry with *trans*‐disposed alkynyl and phosphine ligands. The Pt(1)−P(1) (2.3022(5) Å), Pt(1)−C(1) (2.007(2) Å) and C(1)−C(2) (1.185(3) Å) are all within the usual range of values for complexes of this type,^68^ and display little variation of significance with the electronic character of the alkynyl substituent. In the crystal, the P(1)‐Pt(1)‐C(21)‐C(22) torsion angle (136.6°, −43.4°) prevents any significant extended conjugation through the molecule.

The general structural features of *trans*‐[Ru(C≡CR)_2_(dppe)_2_] complexes have been summarised recently,^65^ and compound **4** offers some points worthy of brief comment. The structure is composed of two independent molecules, both of which are situated on crystallographic inversion centres and which differ in the orientation of the thiophene group. The dihedral angles between the thiophene plane and the plane containing the Ru and C(n1) atoms and the midpoints of the two ligand P atoms are 3.4° for molecule 1, and 96.3 and 77.8° for the two components of the disordered thiophene of molecule 2. Therefore, at least in the crystal, molecules 1 and 2 of **4** are rare examples of such bis(arylacetylide) complexes *trans*‐[Ru(C≡CR)_2_(dppe)_2_] in which the aromatic rings sit close to the “privileged” orientations that allow maximum d–π conjugation along the molecular backbone, and the first in which both conformational isomers are observed for the same chemical compound. The comparable bond lengths within the two molecules are essentially indistinguishable, with the possible exception of the most precisely determined Ru−P bond lengths, which appear to be marginally shorter in molecule 2.

Although the mixed ligand vinylidene–acetylide complex [Ru(C≡CPh){C=C(Me)Ph}{P(OEt)_3_}_4_][CF_3_SO_3_] has been structurally characterised,^66^ compound **5** appears to be the first structurally characterised bis(acetylide) derivative of Ru(C≡CR)_2_{P(OR)_3_}_4_. In the crystal, the tetrakis(triethylphosphite) derivative **5** is situated on a crystallographic $\bar{4}$ axis so that there is only one unique phosphite group. The dihedral angle between the two thiophene groups (which are disordered about the crystallographic twofold axis) is therefore 90°. The Ru−P (2.3149(3) Å) and Ru−C(1) (2.0592(15) Å) distances in **5** are shorter than in the mixed vinylidene–acetylide derivative (Ru−P 2.341(3)–2.350(2) Å; Ru−C 2.114(8) Å) reflecting the increased electron density at Ru in **5** and increased Ru−P and Ru−C back bonding. Although back‐bonding plays only a modest role in the bonding of metal–alkynyl complexes,^69^ the notion is also supported by the trends in C≡C bond lengths in **5** (1.221(2) Å) and the cation [Ru(C≡CPh){C=C(Me)Ph}{P(OEt)_3_}_4_]^+^ (1.209(11) Å). Overall, with the clear exception of the phosphine and phosphite ligands, there are few if any substantive differences in the structures of **4** and **5**.

### Single‐molecule conductance: STM‐BJ and *I*(*s*)

Single‐molecule conductance measurements were carried out by using substrates with a low surface coverage of the molecules of interest on gold substrates. Low surface coverage was chosen to minimise the formation of multi‐molecule junctions and promote formation of single‐molecule events. Adsorption of **2**–**5** at low surface coverage was achieved by immersion of a gold‐on‐glass substrate in CHCl_3_ solutions of the analyte (1 mm) for about 80 s. After adsorption, the samples were washed in ethanol and then blown dry in a stream of nitrogen. All in situ *I*(*s*) and STM‐BJ measurements were conducted in mesitylene, a non‐polar solvent commonly used in STM‐based single‐molecule electrical measurements because of its high boiling point and relatively low vapour pressure. For a given set‐point current and bias voltage, typically 6000–7000 events were observed in both the STM‐BJ and *I*(*s*) experiments.

**Figure 4:**
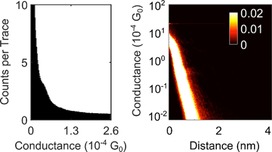
All data 1D (left) and log 2D (right) conductance histograms composed from *I*(*s*) data from **2**. Note the 2D histogram is plotted against units of 10^−4^ G_0_.

Taking compound **2** as a representative example, without any data selection, it is rather difficult to assign a conductance value to the data (Figure 4). Possible reasons for this include a low junction formation probability and short plateau features. The one‐dimensional (1D) conductance histogram of the whole data set shows only a faint shoulder, that is, a conductance peak partially obscured by the exponential background (Figure 4, left). Matching the peak, a faint plateau feature can be seen in the 2D conductance histogram (Figure 4, right), but there is a need for data selection for this system to increase signal‐to‐noise ratio of the data.

Data selection can be made manually, by using a rational criterion, for example by selecting traces with a current plateau that exceeds 0.1 nm in length, and disregarding those without. However, as manual data selection can never be fully objective, it is of interest to compare the results against an automated data selection approach. Here, the unsupervised, automated multi‐parameter vector classification (MPVC) has been adopted to verify the conclusions reached from the manually sorted data.^70^ By way of example, the data for molecule **2** are analysed in more detail in the following paragraphs, which compare the results of manual and automated data selection methods.

For the MPVC, an exponentially decaying current–distance trace was created as a reference vector, *R* (*I*_0_=30 nA, *β*=0.5 Å^−1^). Three vector properties (classifiers) were then calculated for each *I*(*s*) trace with respect to the reference:

1. Δ*X*: the length of the distance vector, *Y*, between reference and *I*(*s*) trace;
2. *θ*: the angle, between *R* and −*Y*;
3. *h*_r_: the reduced Hamming distance, with *h*_r_ being the number of component changes to transform the reduced distance vector, *Y*, into the reduced reference vector, *R*. In this context, “reduced” means that every vector element is divided by its absolute value, so that the resulting vector consists only of 1, 0 and −1.

The whole data set, consisting of 3838 *I*(*s*) traces is thus transformed into 3838 vectors in three‐dimensional space (see cylinder plots in the Supporting Information). Here, similar traces, for example, traces with plateaus, are in close proximity to each other. Plain exponential and plateau‐containing traces are expected to form distinct clusters in this representation and fuzzy c‐means clustering (FCM) was then used to assign the cluster membership.^71^, ^72^ Note that the total number of clusters *k* was selected to be two in this case, to account for plain exponential decays and molecular events, but can be chosen to be a higher number if any cluster consists of sub‐clusters (e.g., to account for a variety of different junction geometries).

**Figure 5:**
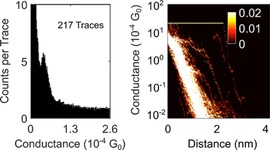
1D (left) and log 2D (right) conductance histograms of cluster 1, containing predominantly *I*(*s*) traces from **2** with plateaus as found by MPVC. These are assigned to “molecular events” (see main text).

**Figure 6:**
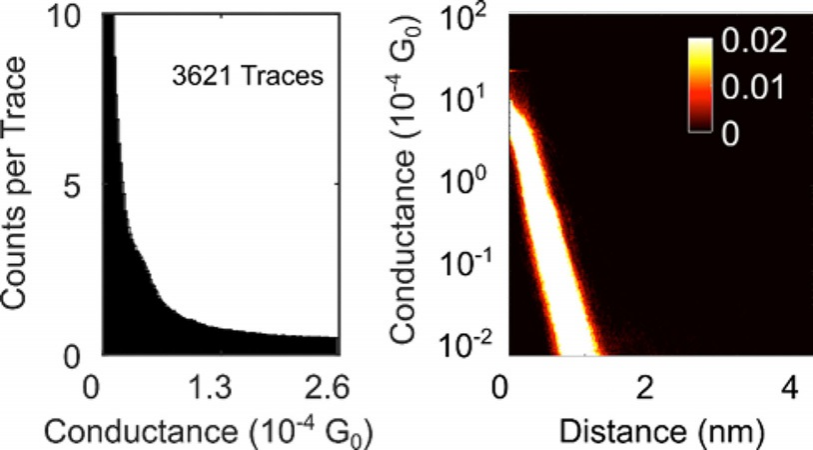
1D (left) and log 2D (right) conductance histograms of cluster 2, containing predominantly plain exponential traces from **2** without plateaus as found by MPVC. These are assigned to *I*(*s*) traces without molecular junction formation.

**Figure 7:**
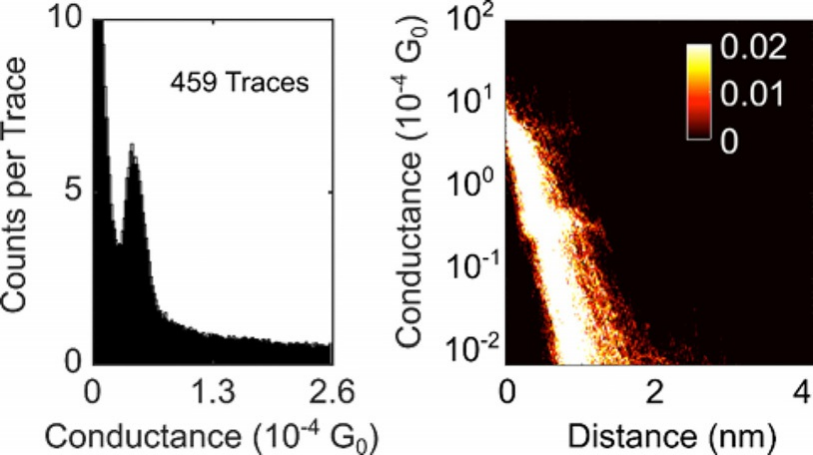
1D (left) and log 2D (right) conductance histograms generated by manual selection of data from **2**. Note that the 2D histogram is plotted against units of 10^−4^ G_0_.

During FCM, 217 (5.65 %) *I*(*s*) traces were assigned to cluster 1, containing predominantly exponential decays with plateaus (“molecular events”, Figure 5). Cluster 2 contains the remaining, predominantly plain exponential traces (3621, 94.35 %; Figure 6). By manual data selection 459 (11.96 %) traces were selected as plateau‐containing (Figure 7).

The results of the unsupervised algorithm approach show excellent agreement with the data selected on the basis of the 0.1 nm plateau length criterion described above in terms of conductances, but with understandable differences in terms of number of selected traces. With respect to the latter, some 81 (37.3 %) of the plateau traces in cluster 1 were also marked as plateau‐containing during the hand‐sorting process. Also, 17.6 % of the manually selected traces were found by the clustering algorithm. The traces found both by MPVC and hand selection are predominantly long plateaus around the most probable conductance value. In addition, 135 traces were included by the MPVC algorithm but not by hand sorting. These traces contained plateaus at various conductance values or unconventional features, meaning deviations from the plain exponential decay other than plateaus. These can possibly originate from different molecular processes during junction formation (or rupture) or noise features. In contrast, some 377 traces were only marked as plateau‐containing during hand selection and not by MPVC. Mostly, those were traces with very short plateau features, or longer plateaus in exponential traces with large decay coefficients. Such features can arise from changes in the molecular junction geometry during the tip retraction process.

After MPVC analysis, cluster 1 exhibits a conductance peak around 0.41×10^−4^ G_0_ (Figure 5). Hand sorting gives a most probable conductance of 0.42×10^−4^ G_0_ (Figure 7). This indicates that although there are differences in the curve selection between hand sorting and automated sorting, the most probable conductance of both data selection methods shows excellent agreement.

While the non‐contact *I*(*s*) technique favours low conductance groups,^1^ the STM‐BJ method generally leads to a greater propensity of higher conductance values. These differences can be explained in terms of the way in which the junctions are formed in both methods. In the *I*(*s*) method, the (typically gold) STM tip is brought into close proximity of the surface to encourage molecular junction formation, but without any initial contact between the STM tip and substrate. In contrast, in the STM‐BJ technique, the STM tip is fused (or crashed) into the substrate and withdrawn to give a metallic filament between the tip and the substrate. Molecular junctions form immediately after the Au–Au point contact breaks.^1^ The rough or fractal nature of these cleaved gold contact junctions often leads to a variety of conductance features in STM‐BJ‐based metal|molecule|metal junctions formed from common anchoring groups such as thiol,^1^, ^73^ carboxylic acid^74^ or pyridine^75^ where in each case more than one single molecule conductance value has been reported, and attributed to differing contact morphologies between the contacting groups and the gold electrode(s).

**Figure 8:**
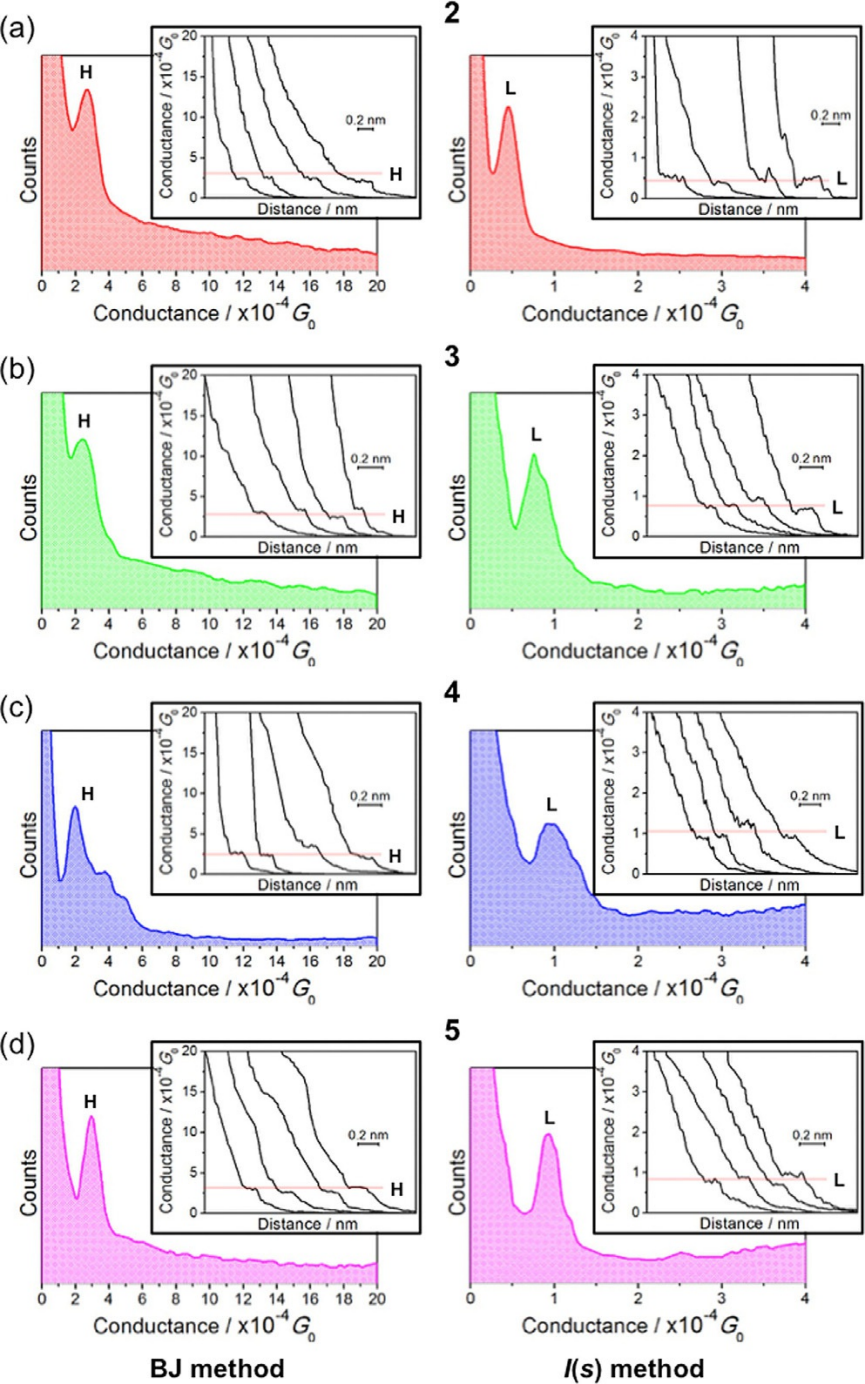
Conductance histograms built by adding all conductance traces (ca. 550) that showed discernible plateaus (with a current plateau that exceeds 0.1 nm in length) as those displayed in the inset of the figures by using either the STM‐BJ (left side) or the *I*(*s*) method (right side). H=high conductance group. L=low conductance group. a) Compound **2**, b) compound **3**, c) compound **4**, and d) compound **5**. Conductance data are referenced to the conductance quantum *G*_0_=2e^2^ h^−1^=77.5 μS. *U*_tip_=0.6 V.

Data recorded by using the STM‐BJ technique for compound **2** at *U*_tip_=0.6 V are summarized in Figure 8. As shown, the conductance profile from these STM‐BJ data for **2** shows only one conductance group (labelled H, for high conductance group). Its most probable conductance ((2.83±0.65)×10^−4^ G_0_, Table 1) is in good agreement with that reported by van der Zant et al. by using the mechanically controlled break‐junction (MCBJ) technique (4×10^−4^ G_0_).^59^

**Table 1:** Single‐molecule conductance data for compounds **2**–**5**.

	Low conductance (L)^[a]^ [G_0_]	High conductance (H)^[b]^ [G_0_]	Break‐off distance [nm]	Calculated (crystallographic) S⋅⋅⋅S distance [nm]
**2**	(0.42±0.10)×10^−4^	(2.83±0.65)×10^−4^	1.70±0.28	1.60
**3**	(0.77±0.14)×10^−4^	(2.70±0.66)×10^−4^	1.85±0.24	1.43 (1.42)
**4**	(1.03±0.28)×10^−4^	(3.18±1.04)×10^−4^	1.90±0.24	1.45 (1.44)
**5**	(0.98±0.14)×10^−4^	(3.12±0.58)×10^−4^	1.90±0.24	1.45 (1.43)

[a] *I*(*s*) method. [b] STM‐BJ method.

As shown in Figure 8 and Table 1, distinct conductance groups were also obtained for the metal complexes **3**–**5** by using the *I*(*s*) (l group, for low conductance group) and the STM‐BJ (H group) method. Interestingly, the compounds **2**–**5** conductance values differ by a factor of about two for the L group, whereas this factor is lower for the H group (Table 1). This demonstrates that the central moiety [C_6_H_4_ vs. [Pt(PEt_3_)_2_] vs. [Ru(dppe)_2_] vs. [Ru{P(OEt)_3_}_4_]) does not exert a great influence on the conductance of these organometallic complexes within these 3‐thienyl contacted Au|molecule|Au junctions.

### Quantum chemical modelling

**Figure 9:**
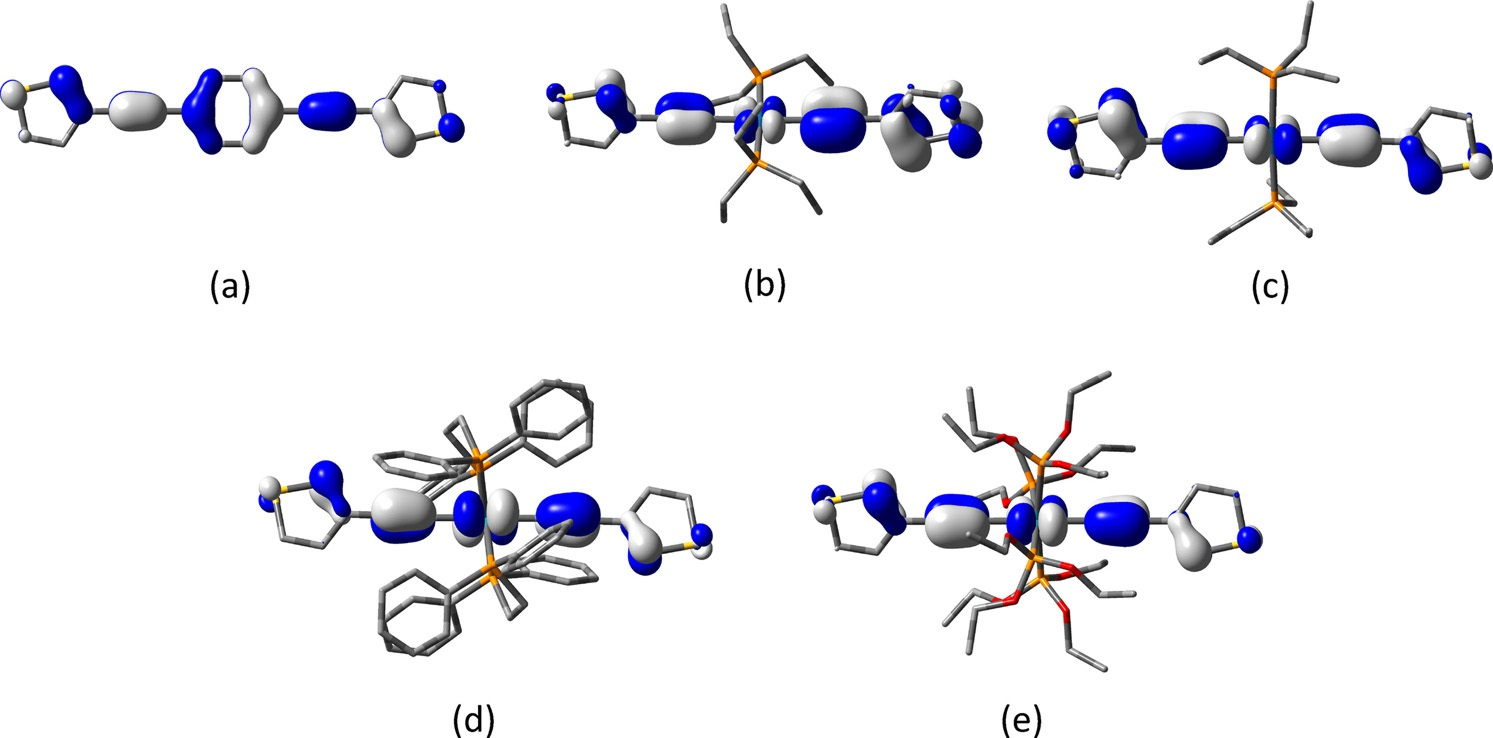
The isosurfaces (±0.04 (e bohr^−3^)^1/2^) of the HOMOs for: a) **2**, b) perp‐**3**, c) planar‐**3**, d) **4**, and e) **5**.

In the quest to better understand the conductance behaviour, the electronic properties of the molecules and electrical behaviour of the junctions have been investigated by using DFT‐based methods. Initial studies of the electronic structures of **2**–**5** were carried out at the B3LYP level of theory^76^ with the LANL2DZ basis set used for metal atoms (Ru, Pt)^77^ and the 6‐31G**^78^ basis set for all other atoms to explore the influence of the central fragment (C_6_H_4_ (**2**), [Pt(PEt_3_)_2_] (**3**), [Ru(dppe)_2_] (**4**), [Ru{P(OEt)_3_}_4_] (**5**)) on the distribution and composition of the frontier molecular orbitals. Plots of the HOMOs are given in Figure 9, and plots of the LUMOs are given in the Supporting Information.

The organic compound **2** again provides a convenient point to commence discussion and a basis for comparison of the metal complexes **3**–**5**. Unsurprisingly, the lowest energy structure features a co‐planar arrangement of the thienyl and 1,4‐phenylene rings, with the frontier orbitals distributed almost evenly across the molecular backbone, making a linear, π‐type conjugated pathway between the two sulfur atoms. For the platinum complex **3**, the lowest energy identified minimum featured the thienyl moieties lying perpendicular to the square plane defining the coordination geometry at the metal centre (perp‐**3**). However, a second minimum, barely 0.8 kcal mol^−1^ higher in energy, in which the thienyl moieties lie in the same plane as the metal coordination sphere (planar‐**3**) was also identified. Again, the HOMOs of these complexes are π‐type and delocalized over the molecular backbone, and feature a small but important metal contribution (perp‐**3**, 10 % Pt; planar‐**3**, 19 % Pt). The LUMOs are more metal in character (perp‐**3**, 42 %; planar‐**3**, 28 %) and rather delocalized in the case of planar‐**3**.

The ruthenium complexes **4** and **5** offer HOMOs that are similarly structured to those described for **2** and offer only marginally more metal character than planar‐**3** (**4**, 33 % Ru; **5**, 24 % Ru). The LUMO of **4** is largely of metal/dppe character, in the case of the phosphite analogue **5** the LUMO is thienyl–π* in character, with the unoccupied metal orbital lying slightly (ca. 0.04 eV) higher in energy.

**Figure 10:**
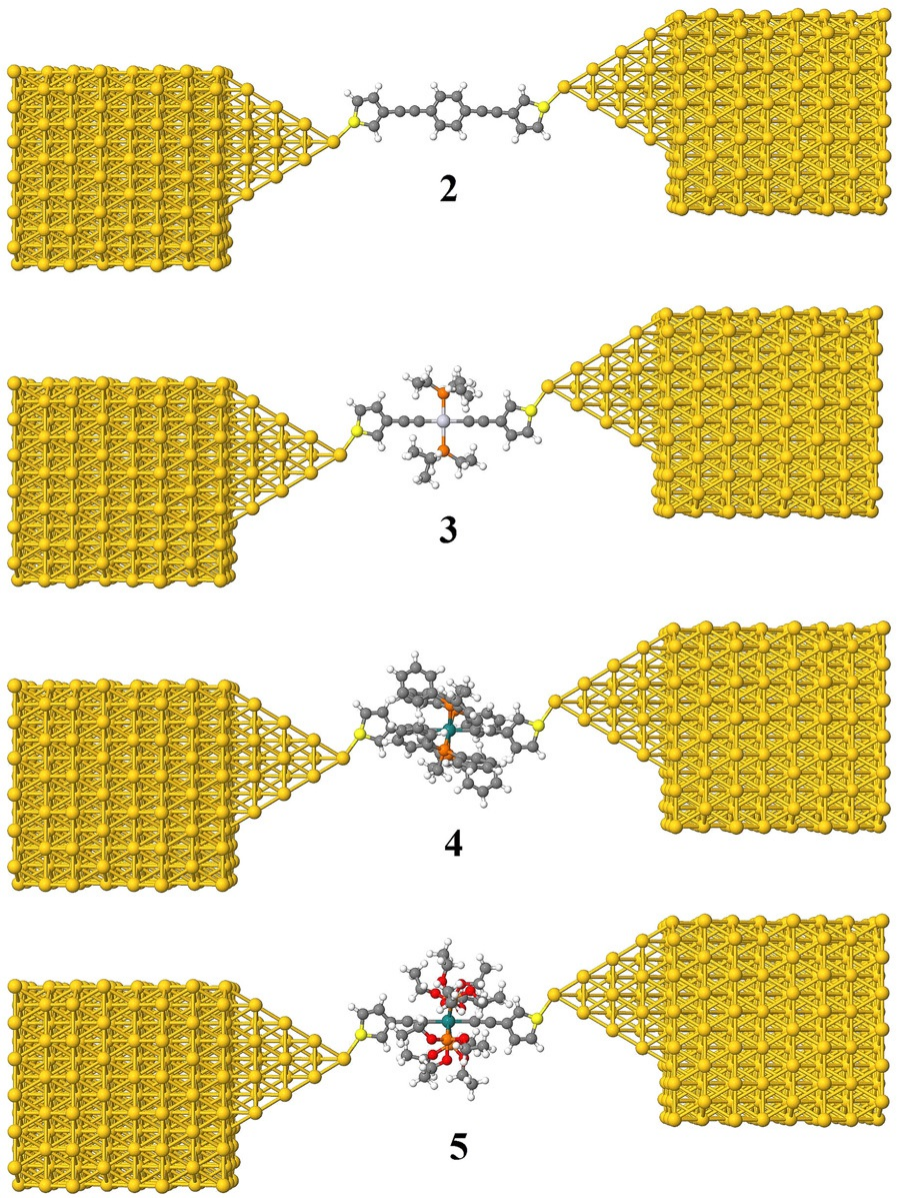
Relaxed geometries of molecular junctions of **2**–**5**.

To provide further insight into the experimentally observed trends, and to better evaluate the properties and behaviour of these molecular junctions, calculations using a combination of DFT (the SIESTA code)^79^ and a non‐equilibrium Green's function formalism were also carried out. For the transport calculations, eight layers of (111)‐oriented bulk gold with each layer consisting of 6×6 atoms and a layer spacing of 0.235 nm were used to create the molecular junctions as shown in Figure 6, and described in detail elsewhere.^80^ These layers were then further repeated to yield infinitely long current‐carrying gold electrodes. Each molecule was attached to two (111) directed pyramidal gold electrodes. The molecules and first layers of gold atoms within each electrode were then allowed to relax again, to yield the optimal junction geometries shown in Figure 10. From these model junctions, the transmission coefficient, *T*(*E*), was calculated by using the GOLLUM code.^80^

**Figure 11:**
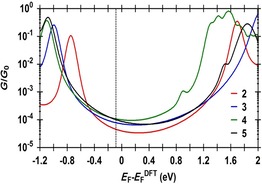
The calculated conductance as a function of the Fermi energy for **2**–**5**. Black dashed line shows the chosen Fermi energy (*E*_F_=−0.075 eV).

A key factor governing the conductance of a molecular junction is the position of the Fermi level of a metal electrode with respect to the molecular HOMO and LUMO levels. In turn, this energy alignment is sensitive to not only the chemical nature of the contacting groups that bind the molecule to the electrode, but also the precise configuration of the metal electrode–molecule contact.^15^, ^81^ However, it is well known that the Fermi energy predicted by DFT (*E*_F_^DFT^) is often not reliable,^33^ and as such the room‐temperature electrical conductance *G* was computed for a range of Fermi energies *E*_F_. The calculated conductances *G* are plotted as functions of *E*_F_−*E*_F_^DFT^ in Figure 11, which reveal similar conductance values over a wide range of Fermi energies, between −0.4 eV to +0.4 eV relative to the DFT‐predicted value. The predicted conductance values of all molecules were compared with the experimental values and a single common value of *E*_F_ was chosen, which gave the closest overall agreement. This yielded a small correction of *E*_F_−*E*_F_^DFT^=−0.075 eV, which has been used in all of the theoretical results described below. Thygesen and colleagues have discussed similar situations for C_60_‐contacted molecular wires, and have shown that critical molecular orbitals can become pinned close to the Fermi level owing to partial charge transfer, leading to good quantitative agreement between calculated and experimentally determined conductance.^82^

The experimental data, now interpreted with the aid of Figure 11, indicates that in all cases the Fermi level lies close to the centre of the HOMO–LUMO gap, but shifted slightly towards the HOMO resonance, and therefore a HOMO‐mediated hole tunnelling mechanism is anticipated in each case.^47^, ^48^, ^49^, ^50^, ^83^, ^84^ However, in contrast to the studies of Wang,^56^ Rigaut^57^ and Mayor^52^ with organic, ruthenium and platinum bis(alkynyl) compounds and complexes contacted into molecular junctions by thiolate groups, conductance values only differing by a factor of ≈2 are obtained across the thienyl‐contacted series **2**–**5**. This lack of variation occurs because although the HOMO and LUMO transport resonances differ significantly between molecules **2**–**5**, transport in the vicinity of the middle of the HOMO–LUMO gap is similar for all molecules (Figure 11).

To further explore the reasons for the small differences in conductance across the series, the nature of the molecule–gold contact was also examined. Table 2 summarises the molecule–gold interaction in terms of the number of valence electrons (*Q*_I_) associated with the molecule, the number calculated on the molecule in the junction (*Q*_MG_) and hence the number of electrons associated with the thienyl SAu contacts (*Γ*) (or “bonds”) based on calculated Mulliken charges. Mulliken charges are basis‐set‐dependent mathematical constructions and therefore only approximately coincide with the physical charge on a molecule. However, it is clear from the data in Table 2 that the value of *Γ*, and the nature of the contact, is only weakly dependent on the nature of the backbone and auxiliary ligands in **2**–**5**. Overall, the molecular conductances of these molecules are similar, with minor variations arising through convolution of the strength of the S→Au bond, and the position of the tail of the HOMO resonance relative to the Fermi level of the electrodes (Table 2).

**Table 2:** The HOMO and LUMO energies of the isolated molecules (eV, c.f. Figure 5), the total number of electrons of the isolated molecule (*Q*_I_), the total number of electrons of the molecule attached to the gold electrodes (*Q*_MG_), the total number of electrons transferred from the molecule (*Γ*=*Q*−*Q*_MG_) and the calculated and experimental *G*/*G*_0_.

Molecule	*E*_HOMO_	*E*_LUMO_	*Q*_I_	*Q*_MG_	*Γ*	Th. *G*/*G*_0_	Ex. *G*/*G*_0_ (L group)
**2**	−5.67	−1.98	94	93.83	0.17	0.45×10^−4^	0.44±0.10×10^−4^
planar*‐* **3** ^[a]^	−4.78	−0.71	164	163.775	0.23	0.77×10^−4^	0.77±0.14×10^−4^
**4**	−4.34	−0.87	350	349.696	0.30	1.06×10^−4^	1.03±0.28×10^−4^
**5**	−4.50	−0.08	322	321.672	0.33	0.99×10^−4^	0.98±0.14×10^−4^

[a] perp‐[**3**] HOMO −4.96 eV, LUMO −0.39 eV.

## Conclusions

The family of 3‐thienylethynyl contacted compounds 1,4‐C_6_H_4_(C≡C‐*cyclo*‐3‐C_4_H_3_S)_2_ (**2**), *trans*‐[Pt(C≡C‐*cyclo*‐3‐C_4_H_3_S)_2_(PEt_3_)_2_] (**3**), *trans*‐[Ru(C≡C‐*cyclo*‐3‐C_4_H_3_S)_2_(dppe)_2_] (**4**) and *trans*‐[Ru(C≡C‐*cyclo*‐3‐C_4_H_3_S)_2_{P(OEt)_3_}_4_] (**5**) have been prepared and studied in metal|molecule|metal junctions by using both STM‐*I*(*s*) and STM‐BJ methods. The compounds **2**–**5** each display two conductance values that differ by a factor of ≈2 within the following range of conductance values: (0.44±0.10–1.03±0.28)×10^−4^ G_0_ (low conductance group) and (2.70±0.66–3.18±1.04)×10^−4^ G_0_ (high conductance group). The MPVC method has been applied to verify the lowest conductance group in an algorithmically definable fashion. For the 3‐thienyl contact employed here, the conductance values obtained by using MPVC and manual data selection were very similar, although there were some differences between the current–distance data sets assigned by each method. The MPVC method, which allows reproducible and objective analysis of conductance features close to the limit of the current amplifier, is therefore a promising avenue for the further exploration of low conductance features. In addition, with an increase in the number of sub‐clusters the method should also prove useful in the analysis of a wider array of junction configurations or in cases where the junction evolves over time or with distance. Further efforts to develop and exploit the MPVC tool are now underway. A quantum chemical analysis of the electronic structures of the isolated molecules reveals a similarly structured HOMO in each case. Within model junctions, the Fermi level lies slightly towards the HOMO resonance in each case, and a non‐resonant hole tunnelling mechanism mediated by the similarly structured HOMOs is proposed. The positioning of the Fermi level well within the HOMO–LUMO gap is proposed to account for the similar conductance behaviour across the series. Our study demonstrates that although for some systems, platinum complexes may well be less conductive than purely organic analogues or similarly structured complexes of the group 8 metals, this is not a universal situation, and by appropriate use of contacts and ancillary ligands to position key molecular orbitals with respect to the Fermi levels of the electrodes, rather efficient molecular wires may be engineered. For the future, it will be of interest to study thermal transport through such wires, as although they have similar electrical properties, their vibrational properties and phonon thermal conductances are likely to differ significantly. This ability to tune the latter, while preserving electronic conductance is an attractive proposition for the design of thermoelectric thin films.^85^

## Experimental Section

### Crystal and refinement data

**3**: C_24_H_36_P_2_PtS_2_, *M*=645.69, monoclinic, *a=*8.6132(1), *b=*11.1767(2), *c=*14.1265(2) Å, *β*=104.958(1)°, *U*=1313.84(3) Å^3^, *T=*180 K, space group *P*2_1_/*n*, *Z*=2, *θ*_max=_36.56°, 31 485 reflections measured, 6229 unique (*R*_int_ *=*0.034), *R*1*=*0.0230 [*I*>2*σ*(*I*)], *wR*2*=*0.0560 (all data), *S*=1.068, Δ*ρ*_max,min_=1.509, −0.775 e Å^−3^.

**4**: C_64_H_54_P_4_RuS_2_ **⋅**CH_2_Cl_2_, *M*=1197.07, triclinic, *a=*10.3342(2), *b=*13.2414(3), *c=*21.4418(5) Å, *α*=78.892(2), *β*=84.176(2), *γ*=71.219(2)°, *U*=2723.36(10) Å^3^, *T=*100 K, space group *P* $\bar{1}$ , *Z*=2, *θ*_max=_34.38°, 63 208 reflections measured, 21 631 unique (*R*_int_=0.033), *R*1*=*0.0464 [*I*>2*σ*(*I*)], *wR*2*=*0.1100 (all data), *S*=1.040, Δ*ρ*_max,min_=1.912, −1.936 e Å^−3^.

The thiophene group on molecule 2 is disordered over two sites with occupancies constrained to 0.5 after trial refinement. Geometries were restrained to ideal values. Both dichloromethane solvent molecules are disordered about crystallographic inversion centres.

**5**: C_36_H_66_O_12_P_4_RuS_2_, *M*=979.96, tetragonal, *a=*11.7879(1), *c=*17.5181(3) Å, *U=*2432.22(5) Å^3^, *T=*180 K, space group *P* $\bar{4}$ 2_1_ *c*, *Z=*2, *θ*_max=_37.64, 49 278 reflections measured, 6324 unique (*R*_int_=0.037), *R*1*=*0.0325 [*I*>2*σ*(*I*)], *wR*2*=*0.0746 (all data). *S*=1.135, Δ*ρ*_max,min_=0.476, −0.356 e Å^−3^.

The thiophene group was modelled as being disordered about the crystallographic twofold axis. One methyl group of the triethylphosphite ligand was also modelled as being disordered over two sites with occupancies constrained to 0.5 after trial refinement.

CCDC 1504230 (**3**), 1504231 (**4**), and 1504232 (**5**) contain the supplementary crystallographic data for this paper. These data can be obtained free of charge from The Cambridge Crystallographic Data Centre.

### Single‐molecule conductance measurements

All single‐molecule conductance measurements were recorded at room temperature in mesitylene with an Agilent 5500 SPM microscope. Molecular adlayers were formed on flame‐annealed gold on glass samples, purchased from Arrandee, Germany. These commercially available substrates were rinsed with acetone and flame‐annealed carefully for about 20 s with a butane torch until a slight orange glow was obtained. This flame‐annealing procedure was performed three times and generally resulted in relatively large area flat Au(111) terraces.^86^ Gold STM tips were fabricated from 0.25 mm Au wire (99.99 %), which was freshly anodically electrochemically etched at +2.4 V for each experiment in a mixture of ethanol (50 %) and HCl (50 %).

Single‐molecule electrical measurements were performed by using both the in situ break‐junction (BJ) and *I*(*s*) methods. The in situ break‐junction method developed by Xu and Tao relies on the formation and cleavage of metallic break junctions between the STM tip and the underlying gold substrate.^63^ Such metallic break junctions are formed by forcing the STM tip a certain distance into the gold substrate. The STM tip is then retracted until the gold–gold contact breaks, which leaves an open nanoscale gap into which the molecular targets can adsorb. These molecular bridges then cleave upon further retraction of the STM tip and molecular conductance can be determined by monitoring the current versus distance retraction profiles.

In the *I*(*s*) technique, a gold STM tip is brought to a fixed distance, determined by the set point conditions, above the gold surface covered with the target molecule under analysis.^62^ Direct metal‐to‐metal contact between the STM tip and substrate is avoided. The initial approach distance of the STM tip to the substrate surface is controlled by the bias voltage and set‐point current (*I*_0_). The measurement involves first locating the STM tip close to the gold substrate at a given height by setting the *I*_0_ and *V*_bias_ values. The feedback loop of the STM is then temporary disabled and the STM tip is rapidly retracted (*s*=distance) while the tunnelling current (*I*) is continuously recorded. At the initial set‐point conditions, the target molecules can be trapped between the STM tip and the gold substrate as a molecular bridge. In such circumstances, during the retraction of the STM tip, the molecular bridge is then pulled up and stretched in the nanojunction until the molecular junction is cleaved. For both the BJ and the *I*(*s*) methods, when the molecular bridge is formed and then cleaved, a characteristic current plateau is typically observed, with a step‐like drop in the current reflecting cleavage of the molecular bridge. On the other hand, if during the tip retraction molecules are not caught in the STM nanogap then the tunnelling current simply decreases exponentially with separation.

Data from the single‐molecule studies are available from the University of Liverpool data catalogue (http://datacat.liverpool.ac.uk/187/).

## Supporting information

As a service to our authors and readers, this journal provides supporting information supplied by the authors. Such materials are peer reviewed and may be re‐organized for online delivery, but are not copy‐edited or typeset. Technical support issues arising from supporting information (other than missing files) should be addressed to the authors.

Supplementary
