# Supplementary material for: Single‐Molecule Conductance Studies of Organometallic Complexes Bearing 3‐Thienyl Contacting Groups
Source: Chemistry. 2017 Jan 16;23(9):2133–43. doi: 10.1002/chem.201604565 (PMC5396322; doi:10.1002/chem.201604565)
Supplement: Supplementary file 1 — Supplementary [file CHEM-23-2133-s001.pdf]

CHEMISTRY
A European Journal

Supporting Information

Single-Molecule Conductance Studies of Organometallic Complexes Bearing 3-Thienyl Contacting Groups

Sören Bock,[a] Oday A. Al-Owaedi,[b, c] Samantha G. Eaves,[a, d] David C. Milan,[e] Mario Lemmer,[f] Brian W. Skelton,[a, g] Henrry M. Osorio,[h, i, j] Richard J. Nichols,[e] Simon J. Higgins,[e] Pilar Cea,[h, i] Nicholas J. Long,[f] Tim Albrecht,[f] Santiago Martín,*[h, k] Colin J. Lambert,*[b] and Paul J. Low*[a]

chem_201604565_sm_miscellaneous_information.pdf

Supporting Information

Multi parameter vector classification (MPVC)

Figure S1 shows all *I(s)* traces in the MPVC representation (ΔX and θ were transformed into Cartesian coordinates). FCM was used to group the *I(s)* traces into two clusters. Marked in red are the curves in cluster 1, in blue are curves of cluster 2.

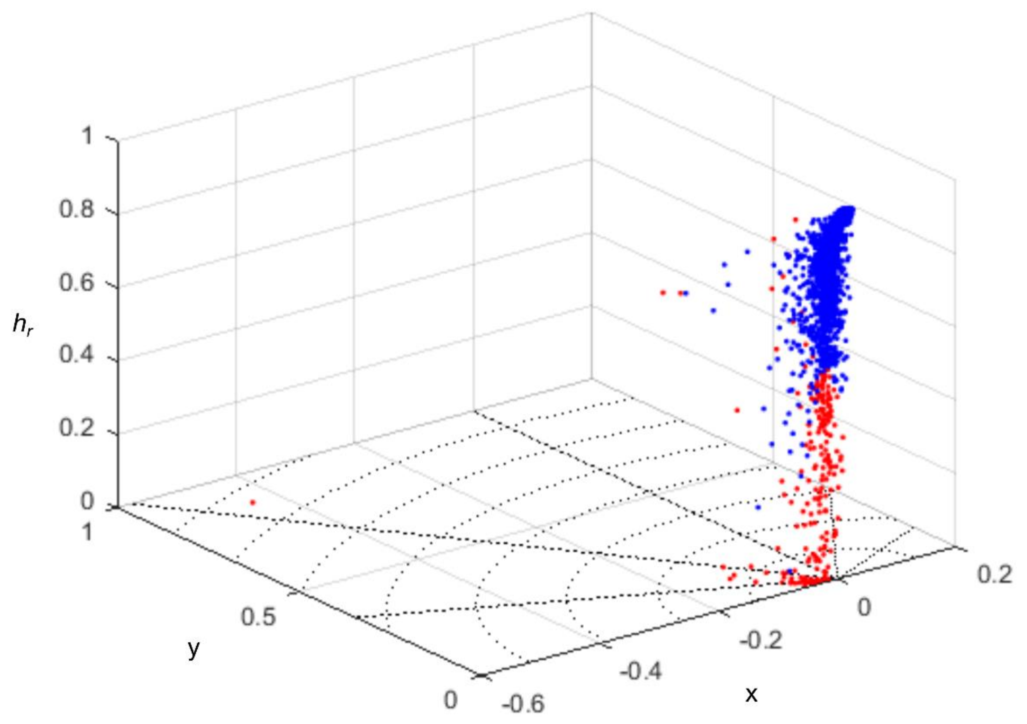

Figure S1: Cylinder plot of the whole data set after MPVC and clustering. Cluster 1 contains the plateau containing traces in red, cluster 2 contains plain exponential traces in blue.

I(s) traces with long plateaus are towards low h_r in the MPVC representation, plain exponential traces towards high h_r. With decreasing plateau length, h_r increases. For the limit of the plateau length approaching zero, the plateau traces are no longer distinguishable from plain exponential traces. Due to the relatively short plateau

features, the plateau traces and plain exponential traces do not form distinct clusters in the MPVC, so that plateaus reaching the length of noise features cannot be distinguished from noisy traces.

With very short plateau features, hand selection has the advantage that, once the plateau current has been estimated, the focus of manual selection can be on a specific part of the curve and thereby enabling to find plateau curves with plateau features of the length of noise features (Figure S2).

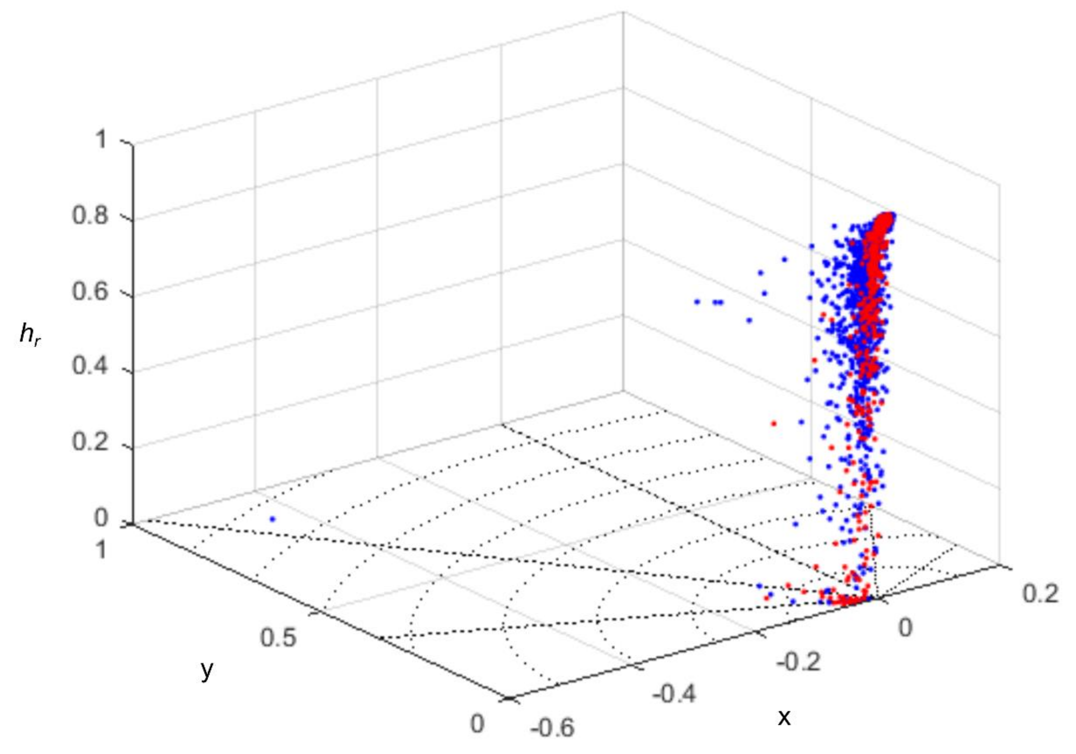

Figure S2: Cylinder plot of the hand-sorted data. Points in red were marked as plateau containing during hand selection process. Close to the origin of the distribution, i.e. towards h_r *of 1 are predominantly traces with short/ without plateaus.*

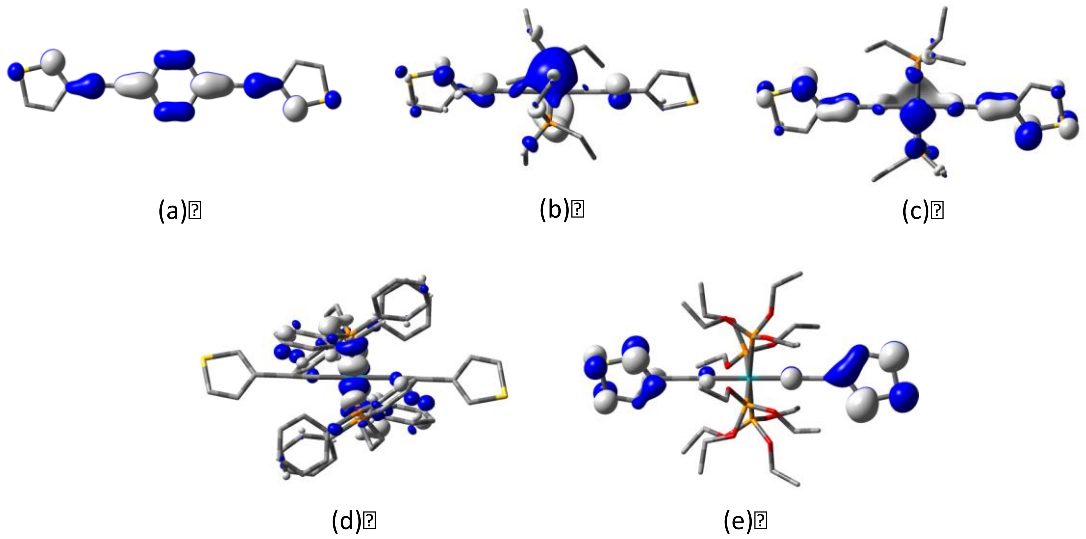

Figure S3. The iso-surfaces (±0.04 (e / bohr^3)$^{1/2}$) of the LUMOs for (a) **2** (b) *perp*-**3** (c) *planar*-**3** (d) **4** (e) **5**.

Synthetic details

General conditions All reactions were performed under an N_2 atmosphere using standard Schlenk techniques. Reaction solvents were purified and dried by appropriate means prior to distillation and storage under nitrogen. No special precautions were taken to exclude air or moisture during work-up. The compounds HC≡C-*cyclo*-3-C_4H_3S (**1**),[S1] 1,4-C_6H_4(C≡C-cyclo-3-C_4H_3S)$_2$ (**2**),[S2] $PtCl_2(PEt_3)_2$ (from a 10 wt % solution of triethylphosphine in hexane),[S3] $Pd(PPh_4)_4$,[S4] [RuCl(dppe)$_2$]OTf[S5] and *trans*-$RuCl_2\{P(OEt)_3\}_4$[S6] were prepared by literature methods, or minor variations. All other materials were obtained from commercial suppliers and used as received.

The NMR spectra were recorded on 400 MHz Bruker Avance, Bruker AV-500 or Bruker AV-600 spectrometers. ^{1}H and $^{13}C\{^{1}H\}$ spectra were referenced to residual protic solvent signals, while $^{31}P\{^{1}H\}$ spectra were referenced to external phosphoric acid. IR spectra were recorded using a Thermo Scientific Nicolet 6700 spectrometer as CH_2Cl_2 solutions in a cell fitted with CaF_2 windows. Electrospray mass spectra were recorded on a Waters LCT Premier. Elemental analyses were performed by London Metropolitan University, London, United Kingdom.

Synthesis of trans-Pt(C≡C-cyclo-3-$C_4H_3S)_2(PEt_3)_2$ (**3**)

A Schlenk flask was charged with dry triethylamine (25 ml), $PtCl_2(PEt_3)_2$ (150 mg; 0.30 mmol) 3-ethynylthiophene (**1**, 76 mg; 0.7 mmol) and CuI (14 mg; 0.08 mmol) and the resulting mixture stirred at room temperature for 7 hours. After removing the solvent under reduced pressure the residue was purified using column chromatography (silica; CH_2Cl_2:hexanes 1:1). The first band was discarded, the second band, containing some mono-substituted by-product was discarded as well and the third yellow band, containing the desired product, was collected. After removing the solvent the product was obtained as pale yellow powder (76 mg; 39 %). IR (CH_2Cl_2) / cm^{-1}: ν(C≡C) 2107 (s). ^{1}H-NMR ($CDCl_3$, 500 MHz) δ / ppm: 1.18 – 1.26 (m, 18H); 2.12 – 2.21 (m,6H); 6.95 – 7.00 (m, 2H, H^2); 7.03 – 7.10 (m, 2H, H^4 or H^5); 7.13 – 7.18 (m, 2H, H^4 or H^5). ^{13}C-NMR ($CDCl_3$, 126 MHz) δ / ppm: 8.52 (s, CH_3); 16.55 (t, J = 17.6 Hz, CH_2), 103.62 (br, C_α or C_β), 106.32 (br, C_α or C_β), 123.57 (s, C^4 or C^5), 124.07 (s, C^4 or C^5), 128.03 (s, C^3), 130.30 (s, C^2).^{31}P-NMR ($CDCl_3$,243 MHz) δ / ppm: 11.46 (s, J_{CPt} = 1186 Hz). EI(+) –MS (m/z): 646 $[M]^+$, 579 $(Pt(PEt_3)_2$(C≡C-thp) + MeCN$]^+$. Anal. Found: C, 44.52; H, 5.71. $C_{24}H_{36}P_2PtS_2$ calc.: C, 44.64; H, 5.62 %.

Synthesis of trans-Ru(C≡C-cyclo-3-$C_4H_3S)_2$(dppe)$_2$ (**4**)

Method A. An oven-dried Schlenk flask was charged with dry DCM (15 ml), [RuCl(dppe)$_2$]OTf (108 mg; 0.10 mmol), 3-ethynylthiophene (**1**, 80 mg; 0.74 mmol) and potassium tert-butoxide (29 mg; 0.26 mmol). After stirring overnight at ambient temperature the dark yellow mixture was filtered through an alumina plug (basic, oven dried). After removing the solvent the crude product was obtained as dark yellow solid, which was recrystallised by slow diffusion of hexane in a CH_2Cl_2-solution (52 mg; 47 %).

Method B. An oven-dried Schlenk flask was charged with dry CH_2Cl_2 (5 ml), DBU (3 drops) and [RuCl(dppe)$_2$]OTf (162 mg; 0.15 mmol). Then, 3-ethynylthiophene (**1**, 108 mg; 1.00 mmol) was added and the mixture was stirred for 5 minutes. To the yellow solution $TlBF_4$ (42 mg; 0.14 mmol) was added and the suspension was stirred for 5 minutes. The yellow mixture was filtered through a alumina plug (basic, oven-dried) and the solvent of the filtrate removed giving the crude product as yellow solid, which was recrystallised by slow diffusion of hexane in a CH_2Cl_2-solution (68 mg; 41 %).

IR (CH_2Cl_2) / cm^{-1}: ν(C≡C) 2060 (s, sh), 2085 (m). ^{1}H-NMR ($CDCl_3$, 500 MHz) δ / ppm: 2.55 – 2.65 (m, 8H, CH_2), 6.50 (dd, J = 3.0, 0.8 Hz, 2H, H^2 or H^4); 6.61 (dd, J = 4.9; 0.8 Hz, 2 H, H^2 or H^4); 6.93 – 7.01 (m, 16H, H^{meta}); 7.09 (dd, J = 4.9; 3.0 Hz, 2H, H^5); 7.14 – 7.21 (m, 8H, H^{para}), 7.49 – 7.57 (m, 16H, H^{ortho}). ^{13}C-NMR ($CDCl_3$, 126 MHz) δ / ppm: 31.36 (q, J = 12.2 Hz, CH_2), 110.55 (s, C_β), 120.09 (s, C^2 or C^4), 122.48 (s, C^5), 126.81 (s, C^{meta}), 128.45 (s, C^{para}), 129.70 (s, C^2 or C^4), 130.16 (s, C^3),

134.21 (s, C^{ortho}), 137.02 (t, J = 10.0 Hz, C^{ipso}) (C_α was not be observed). ^{31}P-NMR ($CDCl_3$,243 MHz) δ / ppm: 53.60. EI(+) –MS (m/z): 1112 $[M]^+$, 1046 $[Ru(dppe)(C{\equiv}C\text{-thp}) + MeCN]^+$, 898 $[Ru(dppe)]^+$. Anal. Found: C, 68.87; H, 4.89. $C_{64}H_{54}P_4RuS_2$ calc.: C, 69.11; H, 4.83 %.

Synthesis of trans-Ru(C≡C-cyclo-3-$C_4H_3S)_2\{P(OEt)_3\}_4$ (**5**)

A Schlenk flask was charged with *trans*-$RuCl_2(P(OEt)_3)_4$ (0.19 g, 0.23 mmol), KPF_6 (0.14 g, 0.77 mmol) and 3-ethynylthiophene (**1**, 0.4 ml, excess), dry degassed EtOH (4 ml) and dry degassed NH^iPr_2 (1.5 ml) and stirred under N_2 for 12 days (or until all *mono*-alkynyl is converted to the *bis*-alkynyl in solution, monitored by unlocked ^{31}P NMR spectroscopy). The solution colour changed from yellow to brown over the reaction period, with a white precipitate observed. After this time, the reaction solution was filtered to remove inorganic salts and the filtrate concentrated to dryness, yielding a brown oily residue. The oily residue was treated with MeOH (3 ml) and left in the fridge for *ca.* 2 h. to aid precipitation of an off-white solid. The solid was collected by filtration and washed with cold MeOH (3 × 10 ml). The solid was then extracted with CH_2Cl_2 and purified by flash chromatography on an alumina (basic, oven-dried) pad. The first pale yellow fraction was collected, yielding a white solid upon removal of the solvent (0.099 g, 45 %). The precipitate may be further purified by recrystallisation from CH_2Cl_2 / EtOH layer diffusion, yielding large white crystals suitable for X-ray crystallography. IR (CH_2Cl_2) / cm^{-1}: ν(C≡C) 2072 (s), 2088 (m). ^{1}H NMR ($CDCl_3$, 600 MHz) δ / ppm: 1.21 (t, J = 7 Hz, 36H, CH_3, $P(OEt)_3$), 4.31 (quar., J = 7 Hz, 24H, CH_2, $P(OEt)_3$), 6.64 (s, 2H, H^4), 6.76 (d, J = 5 Hz, 2H, H^6), 7.05 (t, J = 3 Hz, 2H, H^5). ^{31}P NMR ($CDCl_3$, 600 MHz) δ / ppm: 137.0 (s, RuP_4).^{13}C NMR ($CDCl_3$, 600 MHz) δ / ppm: 16.6 (s, CH_3, $P(OEt)_3$)), 61.0 (s, CH_2, $P(OEt)_3$), 107.4 (s,

C^2), 113.5 (t, J = 20 Hz, C^1), 119.4 (s, C^4), 122.9 (s, C^5), 130.2 (s, C^6), 130.8 (s, C^3).ES (+)-MS (m/z): 980 $[M]^+$, 873 $[Ru(P(OEt)_3)_4(C{\equiv}Cth)]^+$. Anal. Found: C, 43.98; H, 6.73. $C_{36}H_{66}O_{12}P_4RuS_2$ calc.: C, 44.07; H, 6.79 %.

Theoretical Methods

Gas-phase optimizations were performed with the Gaussian 09 program package,[S7] using the B3LYP functional[S8] and LANL2DZ basis set on Ru and Pt [S9] and 6-31G** on all other atoms.[S10] The DFT-Landauer approach used in the modelling of molecular junctions assumes that on the timescale taken by an electron to traverse the molecule, inelastic scattering is negligible. This is known to be an accurate assumption for molecules up to several nm in length.[S11] All molecules in this work have been relaxed in isolation. Geometry optimizations were carried out using the DFT code SIESTA, with a generalized gradient approximation (PBE functional),[S12] double-ζ polarized basis set, 0.01 eV/A force tolerance and a real-space grid with a plane wave cut-off energy of 250 Ry, zero bias voltage and 1 k points.

To compute the electrical conductance, the molecules were then placed in the vicinity of the metal | molecule | metal junctions. Each molecule has been attached to two (111) directed pyramidal gold electrodes. Then, the molecule and the first layer of electrodes were allowed to relax again, yielding the optimal junctions geometries as shown in Figure 10. These layers were then used to extend the gold electrodes to infinity. For each structure, the transmission coefficient $T(E)$ describing the propagation of electrons of energy E from the left to the right electrode was calculated by first obtaining the corresponding Hamiltonian and overlap matrices using SIESTA and then using the GOLLUM code to compute *T(E)* via the relation $T(E) =$

$Tr\{\Gamma_R(E)G^R(E)\Gamma_L(E)G^{R\dagger}(E)\}$, in this expression, $\Gamma_{L,R}(E) = i\left(\sum_{L,R}(E) - \sum_{L,R}{}^{\dagger}(E)\right)$ describes the level broadening due to the coupling between left (L) and right (R) electrodes and the central scattering region, $\sum_{L,R}(E)$ are the retarded self-energies associated with this coupling and $G^R = (ES - H - \sum_L - \sum_R)^{-1}$ is the retarded Green's function, where H is the Hamiltonian and S is the overlap matrix (both of them obtained from SIESTA). Finally the room temperature electrical conductance G was computed from the formula $G = G_0 \int_{-\infty}^{\infty} dE\, T(E)(-\frac{df(E)}{dE})$ where $f(E) = [e^{\beta(E-E_F)} + 1]^{-1}$ is the Fermi function, β=1/k_BT, E_F is the Fermi energy and $G_0 = \left(\frac{2e^2}{h}\right)$ is the quantum of conductance. Since the quantity $(-\frac{df(E)}{dE})$ is a probability distribution peaked at E=E_F, with a width of the order k_BT, the above expression shows that G/G_0 is obtained by averaging $T(E)$ over an energy range of order k_BT in the vicinity of $E = E_F$. It is well-known that the Fermi energy E_F^{DFT} predicted by DFT is not usually reliable and therefore plots are shown of G(G_0) as a function of E_F - E_F^{DFT}. To determine E_F, we compared the predicted values of all molecules with the experimental values and chose a single common value of E_F which gave the closest overall agreement. This yielded a value of E_F - E_F^{DFT} = - 0.075 eV, which is used in all theoretical results.

Crystallography

Crystallographic data for the structure were collected at 180(2) (**3**, **5**) or 100(2) (**4**) K on an Oxford Diffraction Gemini or Xcalibur diffractometer using Mo Kα radiation. Following analytical absorption corrections and solution by direct methods, the structures were refined against F^2 with full-matrix least-squares using the program SHELXL-97.[S13] All non-hydrogen atoms were refined with anisotropic displacement

parameters. All hydrogen atoms were added at calculated positions and refined by use of a riding model with isotropic displacement parameters based on those of the parent atoms.

[S1] D. Solooki, J.D. Bradshaw, C.A. Tessier, W.J. Youngs, *Organometallics* **1994**, *13*, 451-455.

[S2] C.R. Arroyo, S. Tarkuc, R. Frisenda, J.S. Seldenhuis, C.H.M. Woerde, R. Eelkema, F.C. Grozema and H.S.J. van der Zant, *Angew. Chem. Int. Ed.* **2013**, *52*, 3152-3155.

[S3] G.W. Parshall, *Inorg. Synth*. **1970**, *12*, 26-33.

[S4] Coulson, D.R. *Inorg. Synth.* **1972**, *13*, 121-124.

[S5] M.A. Fox, J.E. Harries, S. Heider, V. Perez-Gregorio, M.E. Zakrewska, J.D. Farmer, D.S. Yufit, J.A.K. Howard and P.J. Low, *J. Organomet. Chem.* **2009**, *694*, 2350-2358.

[S6] W.G. Peet, D.H. Gerlach, D.D. Titus, *Inorg. Synth.* **1974**, *15*, 38-42.

[S7] M.J. Frisch, G.W. Trucks, H.B. Schlegel, G.E. Scuseria, M.A. Robb, J.R. Cheeseman, G. Scalmani, V. Barone, B. Mennucci, G.A. Petersson, H. Nakatsuji, M. Caricato, X. Li, H.P. Hratchian, A.F. Izmaylov, J. Bloino, G. Zheng, J.L. Sonnenberg, M. Hada, M. Ehara, K. Toyota, R. Fukuda, J. Hasegawa, M. Ishida, T. Nakajima, Y. Honda, O. Kitao, H. Nakai, T. Vreven, J.A. Montgomery, Jr., J.E. Peralta, F. Ogliaro, M. Bearpark, J.J. Heyd, E. Brothers, K.N. Kudin, V.N. Staroverov, R. Kobayashi, J. Normand, K. Raghavachari, A. Rendell, J. C. Burant, S.S. Iyengar, J. Tomasi, M. Cossi, N. Rega, J.M. Millam, M. Klene, J.E. Knox, J.B. Cross, V. Bakken, C. Adamo, J. Jaramillo, R. Gomperts, R.E. Stratmann, O. Yazyev, A.J. Austin, R. Cammi,

C. Pomelli, J.W. Ochterski, R.L. Martin, K. Morokuma, V.G. Zakrzewski, G.A. Voth, P. Salvador, J.J. Dannenberg, S. Dapprich, A.D. Daniels, Ö. Farkas, J.B. Foresman, J.V. Ortiz, J. Cioslowski and D.J. Fox, Gaussian, Inc., Wallingford CT, 2009.

[S8] a) A.D. Becke, *J. Chem. Phys.* **1993**, *98*, 5648-5652; b) P.J. Stephens, F.J. Devlin, C.F. Chabalowski and M.J. Frisch, *J. Phys. Chem.* **1994**, *98*, 11623-11627.

[S9] a) P.J. Hay and W.R. Wadt, *J. Chem. Phys.* **1985**, *82*, 270-283; b) W.R. Wadt and P.J. Hay, *J. Chem. Phys.* **1985**, *82*, 284-298; c) P.J. Hay and W.R. Wadt, *J. Chem. Phys.* **1985**, *82*, 299-310.

[S10] G.A. Petersson and M.A. Al-Laham, *J. Chem. Phys.* **1991**, *94*, 6081-6090.

[S11] T. Markussen, M. Settnes and K.S. Thygesen, *J. Chem. Phys.* **2011**, *135*, 144104.

[S12] J.M. Soler, E. Artacho, J.D. Gale, A. Garcia, J. Junquera, P. Ordejon, D. Sanchez-Portal, *J. Phys. Condens. Matter* **2002**, *14*, 2745-2779.

[S13] G.M. Sheldrick, *Acta Cryst.* **2015**, *C71*, 3-8.
